# Area-based Deprivation Indices and Healthcare-Associated Infections: A Narrative Review of Evidence

**DOI:** 10.1007/s11908-025-00869-9

**Published:** 2025-10-03

**Authors:** Jacinda C. Abdul-Mutakabbir

**Affiliations:** 1 Department of Pharmacy Practice and Sciences, Skaggs School of Pharmacy and Pharmaceutical Sciences, University of California San Diego, La Jolla, CA 92093 USA; 2 Division of the Black Diaspora and African American Studies, University of California San Diego, La Jolla, CA 92093 USA

**Keywords:** Deprivation index, Social determinants of health, Inequities, Socioeconomic status, Health disparities, Area deprivation index, Social vulnerability index

## Abstract

**Purpose of Review:**

Since the coronavirus disease-19 (COVID-19) pandemic started, there has been a rise in published studies using area-based deprivation indices to explore the link between neighborhood-level social determinants of health (SDoH) and susceptibility to infectious diseases. However, questions remain about how these deprivation indices were developed and how effective they are at identifying and addressing healthcare-associated infection (HAI) disparities. This review aims to clarify the origins of the most commonly used deprivation indices in HAI epidemiology research and to offer key considerations and recommendations for their use to enhance prevention strategies and advocacy efforts.

**Recent Findings:**

The two most frequently used area-based deprivation indices in HAI epidemiology research are the area deprivation index and the social vulnerability index. Of interest, both indices use data from the American Community Survey disseminated by the US Census Bureau to describe area-level socioeconomic and material deprivation across various geographic areas nationwide. Researchers have combined these area-based indices with clinical and individual-level sociodemographic variables and found that higher levels of disadvantage correlate with an increased occurrence of HAIs. Despite similarities in findings when using these indices, they have distinct differences that should be considered.

**Summary:**

Area-level deprivation can increase an individual’s risk of HAIs, and deprivation indices are tools that can quantify this relationship. Despite the availability of relevant data, there is a need to expand the existing literature using deprivation indices in HAI research. Ultimately, this exploratory research has the potential to inform prevention strategies and policy reforms aimed at reducing disparities in HAIs.

## Introduction

Healthcare-associated infections (HAIs) are Infections that occur within 48 h of a hospital admission [1–3]. The most common types of HAIs include catheter-associated urinary tract infections (CAUTIs), central line bloodstream infections (CLABSIs), surgical site infections (SSIs), ventilator-associated pneumonia (VAP), *Clostridioides difficile* infections (CDI), and bloodstream infections caused by methicillin-resistant *Staphylococcus aureus* (MRSA) [3]. According to the Centers for Disease Control and Prevention (CDC), approximately 1 in 31 hospitalized patients has at least one HAI on any given day [2, 3]. In 2018, nearly 1.7 million patients in the US acquired an HAI, resulting in close to 100,000 deaths and significant healthcare costs [1, 2].

Recent studies show that impoverished or disadvantaged populations disproportionately experience higher rates of HAIs, mainly due to systemic inequities in the social determinants of health (SDoH) experienced at the individual and neighborhood levels [4, 5]. Over the past two decades, composite measures have been developed to assess the presence and extent of social and economic disadvantages in different geographic areas across the US [6–8]. These area-level indices have been used both independently and alongside individual measures of deprivation to understand the impact of SDoH on health outcomes [7–9]. Historically, these indices have helped identify health disparities nationwide and to locate areas most vulnerable to disasters, including disease outbreaks [8].

Since the start of the COVID-19 pandemic, there has been a significant increase in research using area-based deprivation indices like the social vulnerability index (SVI) and the area deprivation index (ADI) to examine how social, economic, and material factors at the area level influence infectious disease morbidity and mortality risks [5, 10, 11]. Despite the emergence of this data, questions surrounding the development of the deprivation indices, the applicability of the indices in research on HAI epidemiology, and considerations for the use of the indices to guide HAI prevention strategies remain.

This narrative review aims to synthesize relevant information on the use of deprivation indices in HAI epidemiology research. Specifically, it examines the development of deprivation indices and their application in evaluating the incidence and prevalence of HAIs, highlighting key considerations for their implementation. The review concludes with recommendations for integrating deprivation indices into research, clinical practice (including infection prevention), and policies aimed at reducing equity gaps in the incidence of HAIs.

## Overview of Deprivation Indices

The use of deprivation indices to describe the impacts of area-level circumstances on health outcomes dates back several decades, beginning with the development of the Jarman index [12, 13]. This index was designed to provide a score derived from composite measures related to social, economic, and material deprivation, based on data obtained from the United Kingdom’s census [12–14]. Recognizing the benefits of using spatial epidemiology to identify geographic areas that may be most vulnerable to adverse health outcomes and to allocate resources effectively, the use of deprivation indices expanded across Western countries, including the US. In the US, available indices also rely on census data to compute a single score that reflects area-level deprivation [12, 15–19]. Specifically, the data used in these indices come from the American Community Survey (ACS) [20]. This questionnaire is randomly disseminated across 3.5 million housing unit addresses and group quarters annually by the US Census Bureau [20]. The survey gathers information on a wide range of topics, including income, education, health insurance coverage, and housing costs—supplemental details that are not typically collected in the decennial US census [20].

With the extensive data collected from millions of US households across diverse populations, the ACS can uniquely generate estimates for a wide range of geographic areas [16, 20]. These geographies are organized in a hierarchical structure, with information available at the national, regional, division, state, county, census tract, and block group levels [20]. A more recent addition to the included geographies is information at the ZIP code tabulation area (ZCTA) level, which is a representation of the United States Postal Service ZIP code areas. The hierarchical organization of the geographies is helpful for building deprivation indices that provide valuable insights into how area-level SDoH affects health outcomes. However, it is important to note that data from the most detailed levels tend to be more reliable for outcomes research [8, 16]. Within larger geographical units like states or counties, there can be homogeneity, which may obscure actual SDoH limitations [21]. Conversely, smaller geographic units can better reveal heterogeneity within larger populations and support more targeted responses to SDoH barriers [18, 21, 22]. According to the hierarchical ranking, the US Census Bureau considers census tracts and block groups to be among the smallest geographic units for the ACS [23]. Notably, a census tract generally has a population between 1,200 and 8,000 people, with an optimum size of 4,000 individuals [16, 21, 23]. Census tracts typically cover a contiguous area, and they are developed with the intent of being maintained over long periods to provide a stable set of geographic units for statistical comparison from census to census [20]. Block groups are statistical divisions of census tracts, with a population between 600 and 3,000 people [20, 21]. Each census tract contains at least one block group, and blocks are uniquely numbered within the census tract [20].

### Application of Deprivation Indices in HAI Epidemiology Research

An English literature search for this review was conducted using Google Scholar and PubMed with a combination of search terms such as inequities, disparities, healthcare-associated infections, infection prevention, deprivation indices, social vulnerability index, and area deprivation index. Although the research yielded very few studies that met the criteria, most of the information gathered focused on the two most commonly used publicly available area-level deprivation indices in the US: the area deprivation index (ADI) and the social vulnerability index (SVI) [18, 19]. These findings are discussed in detail in this section. An overview of the indices and the content included in each of them is shown in Table 1.

**Table 1:** Summary of ADI and SVI domains, indicators, geographic level data offerings, and scoring Interpretations

**Area Deprivation Index (ADI)**	
**Domains (Five Theoretical Themes)**	**Indicatiors included (17 total**)
Income	• Median family income (US dollars)^a, b^ • Ratio of Income disparity • Percentage of population below 150% of federal poverty level • Percentage of families below 150% of federal poverty level
Employment	• Percentage of civilian labor force population aged$\:\ge\:$16 years in white collar unemployed^b^ • Percentage of civilian labor force population aged$\:\ge\:$16 years in white collar occupations^a^
Education	• Percentage of population aged$\:\ge\:$25 with < 9 years of education • Percentage of population aged$\:\ge\:$25 with$\:\ge\:$high school diploma^a, b^
Housing	• Median house value (in US dollars)^a^ • Median gross rent (in US dollars)^a^ • Median monthly mortgage (in US dollars) ^a^ • Percentage of owner-occupied housing units ^a^
Household Characteristics	• Percentage of single parent household with children < 18 • Percentage of owner-occupied housing without complete plumbing • Percentage of households without a motor vehicle^b^ • Percentage of household without a telephone • Percentage of households with > 1 person per room
Geographic Level(s) of Data Provided	
Block Groups	
Classification of Data - (Weighted)	
National Percentile Rankings; 1-100(1; least disadvantage and 100; most disadvantage) and State Decile Rankings; 1–10 (1; least disadvantage and 10; most disadvantage)	
**Social Vulnerability Index (SVI)**	
**Domains (Four Theoretical Themes)**	**Indicatiors included (16 total)**
Socioeconomic Status	• Persons 150% below poverty level^b^ • Persons unemployed^b^ • Persons$\:\ge\:$25 years old without a high school diploma^b^ • Housing cost burden • Persons without healthcare insurance
Household composition and disability	• Persons$\:\ge\:$65 years old • Persons < 17 years old • Noninstitutionalized persons > 5 years old with a disability • Single parent household • English Language Proficiency
Housing and Transportation	• Mobile homes • Multi-unit housing structures with more than 10 units • Crowding (> 1 person per room)^b^ • Households without a mobile vehicle^b^ • Persons in group quarters
Minority Status and Language	• Non-Hispanic American Indian/Alaska Native, Asian, African American, Native Hawaiian/Pacific Islander, Hispanic or Latino, two or more races, or another race alone
Geographic Level(s) of Data Provided	
ZIP code Tabulation Area (ZCTA), county, census tract	
Classification of Data - (Unweighted)	
Quartile Rankings (0 to 0.2500; low vulnerability, 0.2501 to 0.5000; low-medium vulnerability, 0.5001 to 0.7500; medium-high vulnerability; 0.7501 to 1.0; high vulnerability)	

^a^Represents the variable that have the greatest weight in the ADI

^b^Represents the variables that are included in both the Area Deprivation Index and Social Vulnerability Index

Table 1 includes a description of the indicators included in the Area Deprivation and Social Vulnerability Indices. It also includes the geographic levels of data that each index provides along with guidance on the classification of the data.

## Area Deprivation Index (ADI)

The ADI, regarded as the most validated deprivation measure in the US, was created to study how neighborhood disadvantage influences health disparities, supporting the development of health interventions and policies that consider these factors [17, 18, 24]. The index was initially developed by the US Health Resources and Services Administration in 2003, and included data extracted from the decennial US Census [17]. The index was modified in 2014 by Kind et al., to incorporate ACS data and to ensure that data were made available at the most granular, block group (600-3,000 individuals), geographical level [18]. The ADI is composed of 17 indicators aggregated into five main theoretical themes: income, employment, education, housing, and household characteristics (shown in Table 1) to create a composite measure of deprivation [18, 21]. The ADI is a weighted index with indicators multiplied by factor score coefficients and summed within the block groups to provide a deprivation score. The indicators related to poverty, income, and education are given the largest weights in the index. As a result of the index weighting, raw scores are not provided to users after a patient’s full address is entered into the tool. The scores can be generated for nationwide or state comparisons and represent the block groups with equal or lower levels of deprivation. The ADI national percentile rankings of block groups range from 0 to 100, with 100 representing the highest level of deprivation [18, 21]. The state rankings for the index range from 0 to 10, with 10 representing the highest level of deprivation [18].

Several studies have employed the ADI to investigate disparities in the incidence and prevalence of common HAIs. In a retrospective analysis of demographic and clinical outcomes in patients with MRSA infections, both community- and healthcare-associated, researchers observed that higher ADI scores correlated with increased odds of healthcare-associated MRSA (HA-MRSA) [11]. They applied both univariate and multivariate analyses to evaluate how individual and area-level factors influence HA-MRSA risk [11]. The authors noted that, in univariate analysis, non-Hispanic Black race was linked to HA-MRSA [11]. However, when ADI was factored into the multivariate analysis, non-Hispanic Black race was no longer associated with higher risk. This suggests race is not a biological risk factor; instead, systemic racism-related community disadvantages, such as lacking social, economic, and material resources, likely contribute to the increased HA-MRSA risk [11, 25, 26].

In a separate retrospective study on pediatric cystic fibrosis patients, researchers geocoded patient addresses using the ADI, rural-urban commuting area codes (RUCA), and other sociodemographic variables like age, race, parental education, and household income, to examine how area-level circumstances contribute to HA-MRSA acquisition [27, 28]. Univariate analysis linked rural residence, low parental education, and low household income with higher neighborhood deprivation, while multivariate analysis found high ADI scores significantly associated with increased HA-MRSA odds [27]. These findings underscore the influence of social factors on HAI risks and highlight how limitations in individual SDoH measures, such as education and income, often coincide with community-level deprivation.

Researchers also used the ADI to explore the link between neighborhood deprivation and the risk of 30-day readmission among Medicare patients diagnosed with CDI [29]. The cohort included all patients from a 20% Medicare claims national random sample who had an inpatient CDI stay between January and November 2024^29^. Unlike prior investigations, the authors separated the cohort using the ADI, with one group representing patients living in the 85% least disadvantaged neighborhoods in the US, and the other in the top 15% most disadvantaged neighborhoods [29]. The results showed that living in the most disadvantaged neighborhoods was linked to a 16% higher chance of readmission [29]. This finding is particularly alarming given that previous research found that 32% of individuals diagnosed with inpatient CDI were readmitted to the hospital, with subsequent hospitalizations characterized by longer lengths of stay and substantial healthcare costs [30]. Consequently, rehospitalization of CDI patients may result in the transmission of the infection to other patients or exposure of the patient to other healthcare-acquired pathogens, which can be disseminated to others [27, 30]. This situation potentially exacerbates health disparities in communities experiencing high levels of social, economic, and material deprivation.

## Social Vulnerability Index (SVI)

The CDC and the National Center for Environmental Health Agency for Toxic Substances and Disease Registry (ATSDR) created the social vulnerability index (SVI) to geographically identify communities at risk of natural disasters and disease outbreaks, supporting their preparedness and response efforts [19]. The index includes 16 indicators collected from the ACS, with data available at the ZIP code tabulation area (ZCTA), county, and census tract levels (shown in Table 1) [19]. Like the ADI, the SVI combines these indicators into thematic categories such as socioeconomic status, household composition and disability, minority status and language, and housing type and transportation to produce a composite score [19]. However, there are essential differences in the areas of focus. A key distinction is the inclusion of minority status and language as a theme in the SVI, acknowledging how systemic racism and exclusionary policies that limit social, economic, and material resources can increase a community’s vulnerability to disasters and disease outbreaks [19, 21]. Also, unlike the ADI, the SVI is not a weighted index, with all indicators having equal weight in the scoring. Percentiles are calculated for each subtheme and the overall SVI after a patient’s address or ZIP code is entered into the tool. The scores can be generated for nationwide or state comparisons, and represent the proportion of tracts, counties, or ZCTAs with equal or lower levels of Social vulnerability. Scores range from 0 to 1, with vulnerability classified into quartiles (0 to 0.2500, 0.2501 to 0.5000, 0.5001 to 0.7500, and 0.7501 to 1.0) and described as low, low-medium, medium-high, and high in terms of vulnerability level, respectively [19].

To a lesser extent, investigators have also used the SVI to observe the link between neighborhood deprivation and the occurrence of HAIs. Of interest, some studies have elected to use only the overall composite SVI (which accounts for all four subthemes) as a surrogate for SES to describe the impacts of neighborhood deprivation on HAI risks. In an ecological study, examining the association between SVI and CDI risks, along with other sociodemographic variables, investigators found that for every 0.1 unit increase in SVI, the rate of CDI increased by 5% [31].

Other studies have examined the association between the overall composite SVI score and subtheme scores with HAI risks. In a retrospective cohort study utilizing National Healthcare Safety Network (NHSN) data and patient addresses, researchers linked both the overall SVI score and its subtheme scores, along with clinical and demographic information, to explore the connection between neighborhood vulnerability and SSI risk [5]. Unsurprisingly, the researchers discovered that patients in the top SVI quartiles had higher odds of developing SSIs compared to those in the lower quartiles after adjusting for clinical and demographic factors [5]. They also observed differences across the subthemes, with patients scoring in the top SVI quartiles for SES and household characteristics facing an increased risk of SSIs; they did not note the same for the race and ethnicity subtheme [5]. However, when the authors stratified the data by race/ethnicity, they did note a more pronounced risk of SSIs in non-Hispanic Black patients when compared to non-Hispanic White patients, albeit not significant [5]. This further emphasizes, through a more direct variable, that neither race nor ethnicity is a risk factor for infection [25, 32, 33]. However, the effects of systemic racism and exclusionary policies like residential segregation or income inequities potentially create area-level barriers to equitable health care for minoritized individuals [33–35].

Noting that area-level deprivation can disproportionately impact minoritized groups, investigators have also utilized the SVI to explore how community-level limitations may contribute to racial differences observed in CDI risks, potentially aiding in the development of early detection and prevention strategies [26, 36]. In a study conducted at an academic teaching hospital, investigators reported that individuals diagnosed with a CDI were more likely to score in the top quartiles of the SVI for both the overall composite score and each subtheme [26]. They also noted that several of the highly vulnerable patients who developed CDI had a visit to the hospital before the CDI diagnosis [26]. Using these findings, the researchers describe potentially collaborating with the institution’s health equity quality improvement team to optimize telehealth and inpatient prevention protocols for patients living in areas with high SVI scores who present with CDI-related symptoms [26].

### The Use of the ADI and SVI to Determine Associations Between Neighborhood-Level Deprivation and Preventable Illnesses that Result in Hospitalization

Preventable viral illnesses are often overlooked factors in developing HAIs. Patients with serious viral illnesses often need hospitalization, which raises their risk of exposure to healthcare-acquired pathogens [37, 38]. It is crucial to recognize how neighborhood-level deprivation affects these infections to better allocate resources and improve infection prevention efforts.

A retrospective study used the ADI to explore the connection between COVID-19 positivity, severity, and inpatient death rates among patients with and without cardiovascular disease [39]. The researchers discovered that individuals from the most deprived areas, regardless of CVD status, were more likely to test positive for COVID-19 or be hospitalized due to the illness [39]. Additionally, a study using the SVI at the ZCTA level to evaluate area-level deprivation and COVID-19 infection found that those with scores in the top quartiles were more likely to be diagnosed with severe COVID-19 and need hospitalization [40]. Similar results were reported by other researchers using the SVI at the census tract level, showing that higher SVI scores across the four subthemes were linked to increased COVID-19 cases [38]. Based on these findings, the authors recommended allocating resources, including personal protective equipment (PPE) and vaccines, to the identified highly vulnerable communities in the area to prevent hospitalizations [38].

## Key Considerations for Using Deprivation Indices in HAI Epidemiology Research

There are essential things to consider when choosing which index to use and how to interpret the results. First, although the ADI and SVI are the most commonly used deprivation indices in the US, especially for HAI epidemiology research, they are not the only tools available to measure area-level social, economic, and material deprivation. The social deprivation index (SDI), also a composite measure of area-level deprivation, was developed by Butler et al. to best direct clinical and community health measures and guide adjustments to quality measures and payments [41–43]. Like the ADI and SVI, the SDI also uses data from the ACS to measure socioeconomic and material deprivation [43]. Unlike the ADI and SVI, the SDI uses seven indicators, with measures provided at county, census tract, ZCTA, and primary care service area (PCSA; small areas of aggregated census tracts developed by Dartmouth University for the Health Resources and Services Administration) [43, 44]. Although this review did not find studies applying this index to HAI epidemiology research, it could be a valuable tool for assessing how neighborhood-level conditions affect HAI susceptibility. Some researchers have also developed other deprivation indices, also utilizing ACS data and other region-specific factors, to measure how socioeconomic disadvantage at the area level influences disease outcomes, which may be worth considering for future HAI research [22, 45]. Different authors have also described using multiple deprivation indices, such as the ADI and SVI together, in their studies to make more comprehensive assessments of the relationships between neighborhood-level deprivation and patient outcomes [46, 47]. This approach may also be helpful for researchers to consider.

Second, although the ADI and SVI have both been used similarly in research, it is important to recognize that they serve different functions and provide data at different geographic levels; therefore, their results may not be interchangeable [21]. The SVI offers data at the ZCTA, county, and census tract levels. Of note, ZCTAs were designed for mail delivery and not for spatial epidemiology analysis [48]. This should be taken into consideration when interpreting the findings reported at this geographic level [48]. Differing from the SVI, the ADI provides data only at the block group level [19, 24]. While block groups combine smaller areas within census tracts, some tract-level data may not be available at the block group level, which could influence data interpretation [16, 20, 21, 23]. Additionally, because the ADI provides information at the most detailed level, a complete address, including the ZIP code, must be entered into the mapping tool [18, 24]. This can be difficult to extract from electronic medical records quickly. With the SVI providing data at larger geographic levels, 5-digit ZIP codes alone can be used to assess vulnerability [19]. For both indices, Geocoding, which is converting the 5-digit ZIP code into a ZIP + 4, or 9-digit ZIP code, can produce more accurate results [20, 21, 49]. Several tools, such as the BC Address geocoder and Geocodio, are available to assist with geocoding addresses; these are browser-based and free services [50].

Third, the ADI is a weighted index that considers poverty, income, and education as the most significant factors [18, 24]. Researchers have observed that the ADI might overemphasize home value because variables are not standardized before ADI scores are computed [51]. Therefore, indicators, like median housing value, which are measured in dollars, are inadvertently given greater weight, meaning that high housing costs could mask other variables and hide the socioeconomic and material hardships of neighborhoods [8, 21, 52]. For example, a study in San Francisco, California, where housing prices are among the highest nationwide, found that the ADI incorrectly classified a disadvantaged area as advantaged [52]. This is likely because housing prices are exceptionally high compared to other regions of the US, leading to a misclassification of disadvantaged areas [52]. Researchers planning to use the ADI in future HAI studies should consider this aspect, as it could impact the populations that are targeted for HAI prevention interventions based on the findings.

Another difference between the indices is the inclusion of race and ethnicity in the SVI, which may be met with scrutiny or legal implications [53]. Race and ethnicity are not variables considered in the calculation of the ADI score [24]. Despite this difference between the indices, investigators have found similar associations when using them to determine the relationship between viral illness incidence, mortality, and area-level deprivation [53].

Furthermore, regardless of the index used, there is a risk of homogeneity within the geographic areas, which could result in the misclassification of a disadvantaged area [21, 43]. In small rural regions (populations less than 600), homogeneity may present a significant challenge, even when data are provided at the block group level [27]. In such cases, it might be helpful to collect individual sociodemographic variables or incorporate rural-based indices alongside the ADI or SVI tools to enhance the assessments.

## Recommendations for Using Deprivation Indices in HAI Research to Improve Clinical, Prevention Strategies, and Promote Policy Changes

The limited amount of research on this topic, as discussed in this review, illustrates how deprivation indices can be used to measure how community-level barriers affect the rate of HAIs; however, there is an urgent need for more research in this area. Additionally, there is a need for scholarly work that explains how these findings can help develop interventions to prevent HAIs, as well as policies that promote widespread access to such preventative measures. This section offers recommendations for using deprivation indices to reduce disparities in HAI rates.

1. **Integrate Area-Based Deprivation Indices alongside Individual Sociodemographic and Clinical Variables to Investigate How Social Factors Can Contribute to HAI Risks.** Historically, research exploring the impacts of social factors on health outcomes has used individual-level sociodemographic variables like age, sex, or race [25, 54]. While the findings from these studies have been helpful, human characteristics like race are not modifiable, which creates difficulty in designing interventions to address the unveiled inequities [11, 33]. As shown by the several studies included in this review, incorporating an area-based deprivation index like the ADI or SVI can provide additional insights into the barriers that patients face due to individual-level sociodemographic factors [5, 11, 26]. Like the studies presented here, the exploratory research examining the association between area-based deprivation and incidence of HAIs will likely be retrospective [5, 11, 26]. Nevertheless, these insights can inform tailored interventions to prevent infections among hospitalized individuals in the most disadvantaged areas.

One example for researchers to consider is from a retrospective study conducted by Diaz et al. [46]. The investigators utilized clinical outcomes and laboratory data, combined with the ADI, SVI, and other sociodemographic variables from the EHR, to predict the presence of AMR organisms in blood cultures for hospitalized patients [46]. Their findings indicated that including the ADI and SVI significantly improved the model’s predictive accuracy. Through this retrospective study, the researchers were able to identify communities at high risk of HAIs caused by AMR pathogens, which may lead to the early screening and deployment of targeted prevention strategies for hospitalized patients from these areas.

2. **Use Research Findings to Guide Interventions That Prevent the Occurrence of HAIs.** There is a gap in the literature regarding the use of research findings on how area-level deprivation affects health outcomes to develop tailored interventions that address identified inequities. However, existing data show how individual-level sociodemographic variables can be used to identify disparities in HAIs and guide the creation of quality improvement protocols. These studies can serve as a reference for clinicians who want to apply area-level deprivation indices similarly.

For example, in a quasi-experimental study conducted by McGrath et al., researchers used sociodemographic factors such as race, ethnicity, and language to identify disparities in CLABSI rates and develop targeted quality improvement strategies [4]. The study found higher infection rates among non-Hispanic Black patients and individuals who speak languages other than English [4]. To address these disparities, the team implemented quality improvement protocols to enhance line check procedures and maintenance for these groups, leading to significant drops in CLABSI rates [4]. Using this as an example, other investigators could adopt a similar approach with SVI or ADI scoring to guide quality improvement efforts for other HAIs like CAUTIs. Notably, the Prioritizing Equity in Antimicrobial Stewardship Efforts (EASE) framework offers detailed strategies, with examples from published literature, on how to create interventions that uncover and reduce inequities in infectious diseases [55]. This framework can serve as a valuable resource to assist clinicians in shaping and improving their methods for addressing HAI disparities.

3. **Advocate for Policies Expanding Access to Preventive Therapeutics to Lower Hospitalization and HAI Risks.** Existing research indicates that some hospitalizations and HAIs could have been prevented through immunizations against preventable viral diseases [56]. Vaccinations are among the most effective ways to avoid hospitalizations caused by infectious diseases, and distributing them within communities at the highest risk for HAIs could serve as a strategic preventive measure [57–59]. Clinicians, scientific organizations, and policymakers must advocate for vaccine access in communities characterized by SVI and ADI scores. While some vaccines are readily available at no cost to socially disadvantaged individuals, others may still be financially out of reach for the communities most in need [60]. Therefore, both public and private payers must recognize the growing need for the coverage of preventive treatments, like vaccines, for residents of high SVI and ADI regions to prevent severe illnesses that could lead to hospitalization. Covering preventable vaccines is also a cost-effective strategy, as the cost of vaccines is significantly lower than the expense of prolonged hospital stays due to HAIs [37].

Allocating vaccines to the most disadvantaged communities may also indirectly decrease the healthcare burden on safety-net hospitals, which serve socially deprived populations, are often underfunded, and frequently face challenges in HAI prevention measures [56, 61]. The decrease in patient load could enable improvements in HAI infection prevention and control strategies [56].

## Conclusion

In conclusion, neighborhood-level SDoH inequities can heighten individual risk for infectious diseases. Using composite measures of socioeconomic and material deprivation, such as the ADI and SVI, in HAI epidemiology research aids in identifying connections between area-level disadvantages and HAI rates. However, it is crucial to understand the specifics of these indices when interpreting results and developing targeted quality improvement and advocacy strategies based on the findings. Ultimately, addressing area-level deprivation is key to reducing disparities in HAIs [61].

## Key References

- Kind AJ, Buckingham WR. Making neighborhood-disadvantage metrics accessible—the neighborhood atlas. The New England journal of medicine. 2018 Jun 28;378(26):2456. 
  - This reference describes the initial development of the Area-deprivation index and further elaborates on the adaptation of the index by Kind et al.
- Centers for Disease Control and Prevention. CDC Social Vulnerability Index. https://www.atsdr.cdc.gov/place-health/php/svi/index.html. 
  - This reference provides background to the development of the social vulnerability index, and it also provides a direct link to the tool for users.
- Rollings KA, Noppert GA, Griggs JJ, Melendez RA, Clarke PJ. Comparison of two area-level socioeconomic deprivation indices: implications for public health research, practice, and policy. PLoS One. 2023 Oct 5;18(10):e0292281. 
  - This reference provides a comprehensive overview of the area deprivation and social vulnerability indices.
- Sood G, Dougherty G, Martin J, Beranek E, Landrum BM, Qasba S, Patel M, Wilson C, Miller A, Sulkowski M, Bennett RG. Is neighborhood deprivation index a risk factor for Staphylococcus aureus infections?. American Journal of Infection Control. 2023 Dec 1;51(12):1314-20. 
  - This reference describes the use of the area-deprivation index to examine the association between area-level disadvantage and healthcare-acquired methicillin-resistant *Staphylococcus aureus* infections
- Dewitt M, Reinke C, Inman M, Bischoff W, Kester S, Neelakanta A, Sampson M, Passaretti C. Exploring social vulnerability in National Health Safety Network surgical site infections. Infection Control& Hospital Epidemiology. 2025 Jun;46(6):589-96. 
  - This reference describes the use of the social vulnerability index to examine the association between area-level disadvantage and surgical site infections.
- Abdul-Mutakabbir JC, Tan KK. Social vulnerability influences racial and ethnic disparities in Clostridioides difficile infection outcomes. Infection Control & Hospital Epidemiology. 2025 Apr 10:1-2.
  - This reference describes the use of the social vulnerability index to further examine racial disparities in *C. difficile* infections at an academic teaching hospital.

## Data Availability

No datasets were generated or analysed during the current study.
